# Accuracy of circulating adiponectin for predicting gestational diabetes: a systematic review and meta-analysis

**DOI:** 10.1007/s00125-015-3855-6

**Published:** 2016-01-14

**Authors:** Stamatina Iliodromiti, Jennifer Sassarini, Thomas W. Kelsey, Robert S. Lindsay, Naveed Sattar, Scott M. Nelson

**Affiliations:** School of Medicine, Glasgow Royal Infirmary, University of Glasgow, Level 2, New Lister Building, Glasgow, G31 2ER UK; Jack Cole Building, University of St Andrews, St Andrews, UK; Institute of Cardiovascular and Medical Sciences, University of Glasgow, Glasgow, UK

**Keywords:** Adiponectin, Gestational diabetes, Meta-analysis, Prediction, Systematic review

## Abstract

**Aims/hypothesis:**

Universal screening for gestational diabetes mellitus (GDM) has not been implemented, and this has had substantial clinical implications. Biomarker-directed targeted screening might be feasible. We sought to determine the accuracy of circulating adiponectin for early prediction of GDM.

**Methods:**

A systematic review and meta-analysis of the literature to May 2015 identified studies in which circulating adiponectin was measured prior to a diagnosis of GDM. Data on diagnostic accuracy were synthesised by bivariate mixed effects and hierarchical summary receiver operating characteristic (HSROC) models.

**Results:**

Thirteen studies met the eligibility criteria, 11 of which (2,865 women; 794 diagnosed with GDM) had extractable data. Circulating adiponectin had a pooled diagnostic odds ratio (DOR) of 6.4 (95% CI 4.1, 9.9), a summary sensitivity of 64.7% (95% CI 51.0%, 76.4%) and a specificity of 77.8% (95% CI 66.4%, 86.1%) for predicting future GDM. The AUC of the HSROC was 0.78 (95% CI 0.74, 0.81). First trimester adiponectin had a pooled sensitivity of 60.3% (95% CI 46.0%, 73.1%), a specificity of 81.3% (95% CI 71.6%, 88.3%) and a DOR of 6.6 (95% CI 3.6, 12.1). The AUC was 0.79 (95% CI 0.75, 0.82). Pooled estimates were similar after adjustment for age, BMI or specific GDM diagnostic threshold.

**Conclusions/interpretation:**

Pre-pregnancy and early pregnancy measurement of circulating adiponectin may improve the detection of women at high risk of developing GDM. Prospective evaluation of the combination of adiponectin and maternal characteristics for early identification of those who do and do not require OGTT is warranted.

**Electronic supplementary material:**

The online version of this article (doi:10.1007/s00125-015-3855-6) contains peer-reviewed but unedited supplementary material, which is available to authorised users.

## Introduction

Gestational diabetes mellitus (GDM) can affect between 1% and 20% of pregnancies, depending on the diagnostic criteria used or the population studied, and is associated with a range of adverse maternal and neonatal outcomes [1–3]. Randomised controlled trials have confirmed that, in routine antenatal care, identification and treatment of GDM, even in its mildest form, reduces the incidence of hypertensive disorders, Caesarean section, macrosomia and shoulder dystocia [4, 5]. Limited resources and infrastructure have, however, led to an ongoing debate regarding the effectiveness of universal screening compared with targeted screening based on maternal baseline characteristics [6], despite the latter identifying only around half of affected women [7].

Improvement of targeted screening by inclusion of first trimester biomarkers may be feasible, facilitating stratified care and early intervention. Yet assessment of fructosamine or HbA_1c_ has not been shown to enhance GDM prediction, due to the substantial overlap of values between affected and non-affected individuals [8, 9]. A range of alternative biomarkers have been proposed: the adipokine adiponectin (measurable in the non-fasting state) is the most promising [10], as it seems to nicely ‘capture’ insulin resistance, a precursor of the disease, rather than measure glycaemia per se [10]. Contemporary cross-sectional studies have shown that GDM is associated with lower levels of circulating adiponectin [11], but the majority of studies assessing the value of serum adiponectin as a screening tool prior to the diagnosis of GDM are limited by their small sample size [12, 13], or restricted to specific subpopulations [13–15]. To provide a more accurate estimate of the effect size we undertook a systematic review and meta-analysis of all eligible studies to examine whether adiponectin can be a useful early pregnancy predictor of future GDM.

## Methods

The study was conducted according to the PRISMA guidelines [16], and followed a structured protocol which was agreed among the authors in advance of the literature search.

### Data sources and searches

We searched several electronic databases up to May 2015, namely, PubMed, EMBASE, MEDLINE, Web of Science and the Cochrane Library. The following search terms were combined using Boolean rules: gestation*, diabetes, pregnan*, adiponec*, with a filter for human studies without language restriction. The full search strategy is provided in the electronic supplementary material (ESM Methods). Two researchers (SI and JS) screened all the titles and abstracts; studies including data on circulating adiponectin levels and GDM were read in full. The reference lists of the selected papers were hand-searched to identify additional papers. Grey literature was searched via the open-grey website.

### Study selection

We included studies that met the following criteria: (1) the study population included women of reproductive age without existing diabetes; (2) serum adiponectin was measured prior (pre-pregnancy or index pregnancy) to the diagnosis of GDM; (3) the clinical outcome was GDM diagnosed by OGTT against reference criteria (in view of the chronological evolvement of the diagnostic criteria and discrepancies across different countries, we did not restrict to specific diagnostic criteria); (4) any study design, apart from case reports, without a language restriction. In the meta-analysis, we included studies if 2 × 2 tables could be constructed from published or requested data.

Two researchers (SI and JS) independently assessed the papers for final selection. If a study fulfilled the eligibility criteria, it was included in the systematic review. Any discrepancies were resolved with discussion. A third reviewer (SMN) was consulted if any unresolved issues persisted.

### Data extraction and quality assessment

If a study had extractable data to construct a 2 × 2 table with the number of false positives, false negatives, true positives and true negatives, or the sensitivity, specificity or AUC of the receiver operating characteristic (ROC) curve of circulating adiponectin in the prognosis of GDM, then it was included in the meta-analysis. If a study was selected for the systematic review but did not provide data that could be included in the meta-analysis, the authors were contacted via e-mail. If the authors did not reply or did not provide the requested information, but the relevant information was extractable using a reverse engineering technique through Plot Digitizer (downloaded from http://plotdigitizer.sourceforge.net/), a computer software programme which can extract data from published plots, the articles and data therein were used in the meta-analysis [17–19].

We developed a data extraction Excel sheet which included the following information: study characteristics (first author, year of publication, number of participants with and without diagnosed GDM), study sample characteristics (mean age, BMI, ethnicity if applicable), test characteristics (method of measuring serum adiponectin, mean value of adiponectin in those with and those without GDM, threshold levels, sensitivity, specificity and AUC of ROC if available in the primary studies) and reference standards for diagnosing GDM.

We used the QUADAS-2 checklist to assess the quality of the selected studies and the risk of bias associated with the study protocol [20]. We assessed the risk of publication bias and potential small study effect visually by constructing a funnel plot, which plots estimates of diagnostic accuracy against statistical precision [21]. In addition, we performed a linear regression of log diagnostic ratios on the inverse root of effective sample sizes as a test for funnel plot asymmetry, where a non-zero slope coefficient (*p* < 0.10) is suggestive of asymmetry and small study bias [22].

### Data synthesis and analysis

We used STATA/SE (version 12.1; StataCorp LP, College Station, TX, USA) and SAS/STAT software (SAS Institute, Cary, NC, USA) for statistical analysis. We used the random effects model for binary data to estimate a summary measure of diagnostic odds ratio (DOR) with 95% CIs. The DOR summarises the diagnostic accuracy of a test and can take values from 0 to infinity. A DOR of 1 represents an uninformative test; as the DOR increases it reflects a test with increasing discriminatory power. Herein, it expresses the odds of having low levels of adiponectin below a given threshold associated with GDM in each study (positive test results) among women with GDM relative to the odds of high adiponectin levels among women without GDM [23, 24]. Heterogeneity resulting from true diagnostic accuracy not being identical in each study was quantified by the *I*^2^ measure [25], and visually explored by generating a forest plot for the DOR of adiponectin with 95% CIs for each individual study. We conducted meta-regression analysis to assess what proportion of the heterogeneity is explained by the discrepancy in specific variables (BMI, age, method of measuring adiponectin, reference criteria of diagnosing GDM, adiponectin cut-off points, timing of measuring adiponectin and ethnicity) among the pooled studies and sensitivity analysis to assess the impact of differential timing in measuring adiponectin and the impact of different participant characteristics in our pooled estimates. We adjusted the DOR separately for age, diagnostic criteria of GDM and BMI. In addition, we estimated a summary ROC curve, specificity, sensitivity, and negative and positive likelihood ratio of adiponectin in predicting GDM by fitting a two-level mixed logistic regression model, with independent binomial distributions for the true positive and true negative restricted to the sensitivity and specificity in each study, and a bivariate normal model for the logit transforms of sensitivity and specificity between studies [26–28].

### Ethics

Ethics approval was not required, as we pooled previously published studies.

## Results

### Search results

**Fig. 1:**
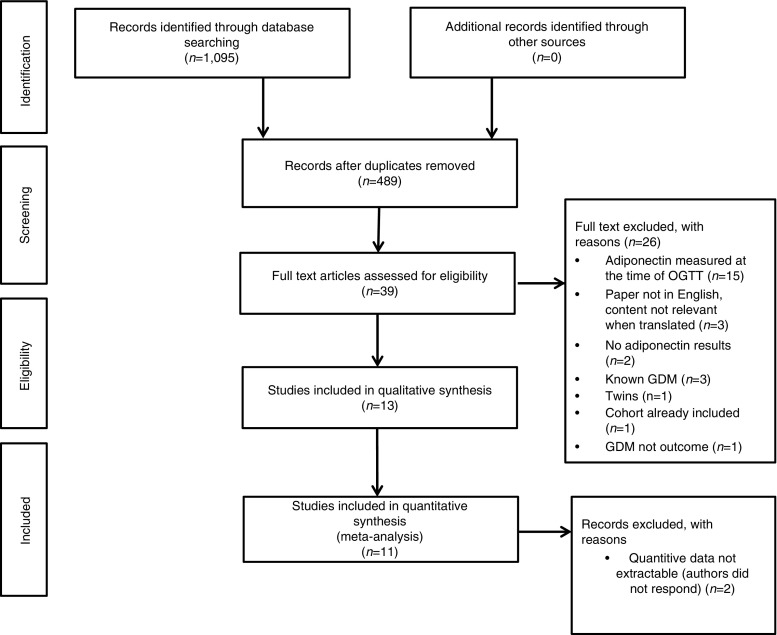
Flowchart of the systematic search methodology

Figure 1 summarises the search process and numerical selection of the final papers that were included in the systematic review and meta-analysis. The systematic search of the biomedical databases resulted in 1,095 hits; after excluding duplicates, 489 citations were identified. We did not identify any unpublished literature relevant to our topic. We selected 39 papers based on their abstract and title, which were read in full for eligibility. Two eligible studies referred to the same study group; hence, only one of them was included in the systematic review [29, 30]. Thirteen individual studies fulfilled the eligibility criteria and were included in the systematic review [12–15, 29, 31–38], whereas 11 studies had extractable data after contacting the authors and were included in the meta-analysis [12–15, 29, 31, 32, 34, 36, 37].

### Description of studies

The characteristics of the 13 studies included in the systematic review are listed in ESM Table 1. The majority of the studies assessed the value of adiponectin in the first trimester, whereas the diagnosis of GDM was established at the end of the second trimester by an OGTT [12–14, 31, 33–37]. Some studies sampled specific populations such as nulliparous women [31], women at high risk of developing GDM [13, 15, 32, 35], or specific ethnic groups (Chinese [33] or Thai [32]). Almost half of the studies used the 100 g OGTT and American Diabetes Association reference criteria for the diagnosis of GDM [13, 14, 31, 32, 35, 38]; others used the 75 g OGTT and the International Association of the Diabetes and Pregnancy Study Groups reference criteria [15, 36], or small variations of them [12, 37], or the 1999 WHO criteria [29, 34].

### Quality assessment

ESM Fig. 1 outlines the methodological quality of the selected studies assessed by the QUADAS-2 tool. The majority of the studies were ranked as high quality for most of the domains.

### Meta-analysis

**Fig. 2:**
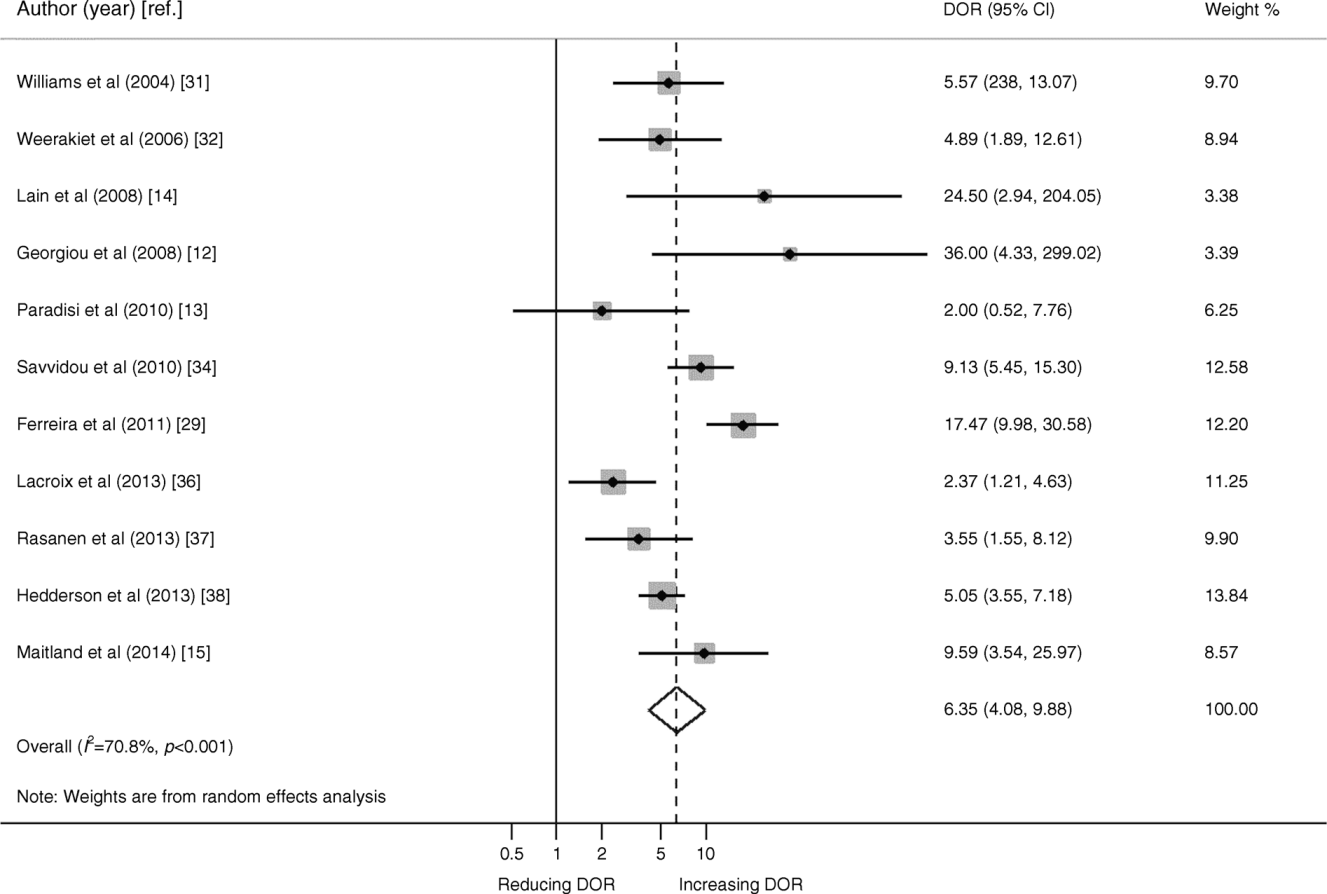
Forest plot of DOR of the studies included in the meta-analysis

We present data from 11 studies with extractable data incorporating 2,865 women of whom 794 were diagnosed with GDM by OGTT following adiponectin testing. Figure 2 shows the study-specific and summary DOR for serum adiponectin predicting GDM later in the index pregnancy among women undergoing adiponectin testing prior to OGTT. The pooled DOR was 6.4 (95% CI 4.1, 9.9). After adjustment for age, the summary DOR did not change substantially (6.4 [95% CI 4.2, 9.8]). After adjustment for different diagnostic criteria of GDM, the pooled DOR was 6.3 (95% CI 3.3, 12.0). After adjustment for BMI the summary DOR was 6.4 (95% CI 4.2, 9.8).

**Fig. 3:**
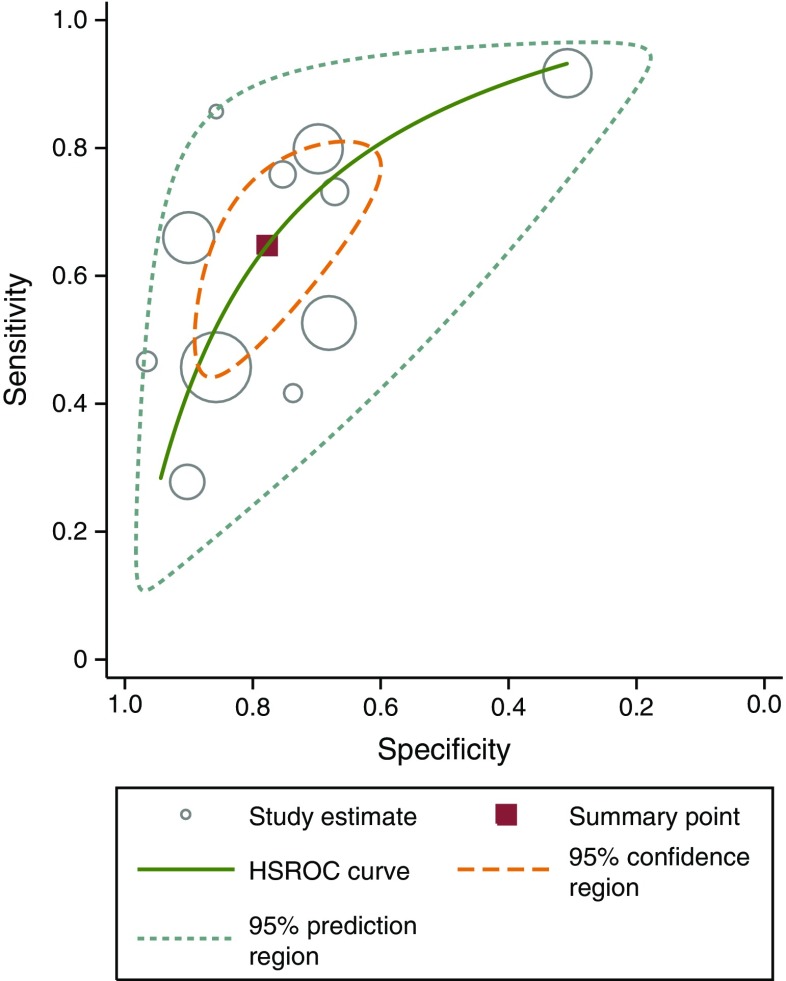
HSROC curve of circulating adiponectin in the prediction of GDM with 95% confidence region and 95% prediction region. The AUC is 0.78 (95% CI 0.74, 0.81)

Figure 3 summarises the pooled sensitivity and specificity as a summary ROC. The pooled sensitivity of predicting GDM with the means of circulating adiponectin was 64.7% (95% CI 51.0%, 76.4%) and the pooled specificity was 77.8% (95% CI 66.4%, 86.1%). For diagnosis of GDM, the positive likelihood ratio of adiponectin testing was 2.9 (95% CI 2.1, 4.1) and the negative likelihood ratio was 0.45 (95% CI 0.34, 0.61). The AUC of the ROC was 0.78 (95% CI 0.74, 0.81). ESM Table 2 shows the corresponding negative and positive predictive values for different prevalence points of GDM. For a prevalence of 10% the corresponding negative predictive value is 95.2% and the corresponding positive predictive value is 24.5%.

### Sensitivity analysis

The majority of the pooled studies measured circulating adiponectin during the first trimester, with the exception of the study by Weerakiet et al [32], which measured serum adiponectin between 21 and 27 weeks; the study by Hedderson et al [38], which refers to pre-pregnancy circulating levels of adiponectin; and the study by Maitland et al [15], which measured adiponectin at 15^+0^–17^+6^ weeks’ gestation. Sensitivity analysis of the remaining studies that assessed the value of adiponectin in the first trimester resulted in a sensitivity of 60.3% (95% CI 46.0%, 73.1%), a specificity of 81.3% (95% CI 71.6%, 88.3%) and a DOR of 6.6 (95% CI 3.6, 12.1). The AUC was 0.79 (95% CI 0.75, 0.82), which was not substantially different from the summary estimates that included all 11 studies, but with greater CI because of the smaller sample size.

Three studies included patients at high risk (based on BMI, previous history of GDM, family history of diabetes) of developing GDM [13, 15, 32]. Sensitivity analysis of the studies that assessed the predictive accuracy of circulating adiponectin in non-high-risk patients resulted in a sensitivity of 59.7% (95% CI 45.7%, 72.2%), a specificity of 82.5% (95% CI 73.5%, 88.9%) and a DOR of 7.0 (95% CI 4.1, 11.8). The AUC was 0.79 (95% CI 0.76, 0.83). The summary DOR of circulating adiponectin for patients at high risk of developing GDM [13, 15, 32] was 5.0 (95% CI 2.2, 11.3). The hierarchical model for estimating summary sensitivity and specificity could not be computed because of the small number of studies.

### Sources of heterogeneity

The estimated *I*^2^ value was 70.8%. Univariate meta-regression analysis showed that different age of women among the studies explained 57.9% of the heterogeneity. Ethnicity distribution within the studies accounted for 59.4% of between-study heterogeneity. Multivariate meta-regression demonstrated that both covariates (ethnicity and age) accounted for 100% of between-study heterogeneity. The different methods of measuring adiponectin accounted for 5.4% of the between-study variation in the DOR, yet there was no suggestion of individual test superiority (*p* = 0.28). Between-study differences in the BMI, the threshold of adiponectin for discriminating women with GDM from women without GDM, the reference criteria of OGTT for diagnosing GDM, the timing of the adiponectin test or the study design had a negative *r*^2^, which suggests that each covariate explained less of the heterogeneity than would be expected by chance. The funnel plot shown in ESM Fig. 2 visually goes against clear evidence of bias by small sample size (*p* = 0.77).

## Discussion

Our study suggests that early pregnancy measurement of circulating adiponectin in isolation has a moderate predictive accuracy for the development of GDM and could facilitate targeted OGTT screening [39]. This would be particularly relevant to areas where universal screening for GDM is not implemented, as circulating adiponectin could be measured inexpensively in the routine non-fasting first trimester bloods, and should improve discrimination of those who will require OGTT in the second trimester from those who will not. Hence, adiponectin testing has the potential to minimise the number of negative resource-intense OGTTs and increase the accuracy of the risk factor approach for GDM screening, eliminating the number and health implications of false-negative cases. In addition, early pregnancy adiponectin may facilitate stratified care, ensuring early identification of women at high risk of GDM, targeted early interventions and prevention of overt GDM development.

This is the first study assessing the summary predictive value of low circulating adiponectin predating the diagnosis of GDM. Low adiponectin has been linked with type 2 diabetes: a 1−log μg/ml increase in serum adiponectin was associated with a 38% decrease in the risk of developing type 2 diabetes [40]. Circulating adiponectin has been extensively studied in GDM in over 20 studies [10]. A recent meta-analysis demonstrated that patients with GDM have substantially decreased adiponectin levels compared with those without GDM [11]. However, we are unaware of any study assessing its summary prognostic accuracy for the early identification of women who will or will not develop GDM, a much more meaningful question since, unlike the non-pregnant situations where risk scoring can be easily followed by fasting glucose or HbA_1c_, GDM diagnosis requires the much more cumbersome and costly OGTT. Thus, any simple test which can meaningfully lessen this burden has potential to improve clinical practice.

Adiponectin is an adipocyte-derived hormone whose circulating levels are closely and inversely related to insulin concentration and insulin resistance, and is a good reflection of whole body insulin sensitivity [41]. It is also inversely associated with BMI, intra-abdominal fat, atherogenic lipid profile, hyperglycaemia, insulin resistance and type 2 diabetes in non-pregnant women [42]. While there is debate about the direction of causality in these relationships, adiponectin has undoubted utility in defining more or less insulin-resistant groups, consistent with evidence that prognostic factors are not required to be causally associated with the outcome of interest. We postulate that women who are more insulin resistant and hence have a concomitant low level of adiponectin (due to hyperinsulinaemia) outside pregnancy become more insulin resistant in pregnancy and are more likely to develop GDM. That lower levels of adiponectin outside pregnancy are associated with a higher risk of GDM in a subsequent pregnancy [38] supports this hypothesis and renders adiponectin a biological plausible prognostic factor for GDM. That the AUC of adiponectin for GDM of 0.78 (95% CI 0.74, 0.81), and 0.79 (95% CI 0.75, 0.83) if it is measured exclusively in the first trimester, exceeds the performance of adiposity measures for prediction of type 2 diabetes (AUCs for single adiposity measures 0.66–0.73), and is equivalent to the AUCs for glycaemia variables including fasting glucose (AUCs 0.73–0.78), suggests that it could be used in a similar fashion to these for stratification of risk. Furthermore, adiponectin has the additional substantial advantage of being measurable in non-fasting samples [43].

Notably, combinations of multiple conventional risk factors in early pregnancy (ethnicity, BMI, family history of diabetes, obstetric history) often do not predict later GDM well. A Health Technology Assessment systematic review found that risk factors as a screening test produced sensitivities of 50–69% and specificities of 58–68% [7]. Measures of glycaemia in early pregnancy also performed relatively poorly: for example, fasting glucose has previously been shown to have an AUC of 0.62, with a sensitivity of 47% and a specificity of 77% [44]; HbA_1c_ has a sensitivity of 19% and a specificity of 95%; and fructosamine has a sensitivity of 12% and a specificity of 95% [9]. In that context, the current data suggest that adiponectin is a reasonably good predictor, even after adjustment for BMI and age. It would appear very important to further investigate the role of adiponectin in early pregnancy as a clinical tool. First trimester circulating adiponectin has the potential to improve the discrimination between ‘high’ and ‘low’ risk women for developing GDM, and facilitate targeted screening for GDM. Furthermore, adiponectin performs well in women who are deemed ‘low risk’ based on conventional risk factors (DOR 7.0 vs 5.0 in ‘high risk’) and may improve reclassification. Given the discrepancy in the prevalence of GDM within the different populations studied, the performance of adiponectin in women at ‘high risk’ and women at ‘low risk’ should be validated in prospective cohort studies.

### Strengths and weaknesses of the study

This is the first study presenting pooled data in a large number of women to assess the predictive value of circulating adiponectin in early identification of women who subsequently develop GDM. The strengths of this review lie in its methodology. We used an extensive search strategy [24], did not use a language restriction, contacted the authors when data were not extractable, and used robust statistical analysis in line with established guidance for diagnostic tests [45, 46]. Although, the process of systematic review and meta-analysis is a robust way of estimating the true effect size, with less random error because of increased sample size, the inferences estimated by the pooled data are subject to the limitations of the primary studies. Between-study heterogeneity may be self-limiting in pooling studies together to estimate a summary measure. In the current meta-analysis, heterogeneity appeared to be mainly attributable to the variation in age and ethnic distribution across different studies, but the degree of heterogeneity (*I*^2^ was 70.8%, suggesting a moderate degree of heterogeneity [25]) and the consistency in the direction of effect rendered it acceptable to pool studies together. We used optimal methodology to tackle the limitation of heterogeneity in the meta-analysis, since the hierarchical summary ROC (HSROC) analysis takes into account the full range of variation in the data differentiating within study from between study and systematic from random variability [47]. In addition, we conducted a meta-regression analysis to investigate the sources of heterogeneity among the results of each study and used random effects analysis, rather than fixed effect, which incorporates unexplained heterogeneity among studies [24]. Our systematic review included 13 studies of generally high quality. Although two studies did not have extractable or available data, even after contacting the authors [33, 35], we do not expect that this will have introduced substantial bias in our pooled estimates, since they were relatively small studies (*n* = 42 [33] and *n* = 32 [35]) and both showed a significant association between low adiponectin and GDM; hence, we anticipate that their point estimates would have been in the same direction with our pooled estimate. In addition, a proportion of the patients of the study by Iannielo et al [35] had been included in a previous study [13] which was part of our meta-analysis. We acknowledge that the summary sensitivity and specificity need to be interpreted and quoted with caution, as individual studies did not have identical thresholds of adiponectin to discriminate between those at high and those at low risk of GDM, and summary estimates of sensitivity and specificity can vary according to the threshold used. However, we confirmed that the different cut-off points of adiponectin did not attribute to the between-study heterogeneity in the point estimates. We also quoted the summary DORs in each analysis, which is generally constant regardless of the diagnostic thresholds used [48]. Our study did not suggest a summary threshold of adiponectin, as this would be inappropriate given the different patients’ characteristics, including ethnic distribution, and the variation in the methods of measuring serum adiponectin. We acknowledge that the majority of the studies included individuals from different ethnic backgrounds: given the different thresholds of obesity within different ethnic groups and the lack of ethnic stratification in BMI within each study, adjustment of the summary estimates for BMI may not have accounted for the magnitude of the ethnic variation in BMI. An additional limitation of our meta-analysis is that the majority of the pooled studies were of case–control design, which can potentially inflate the point estimate when pooled together [49]. However, most were derived from retrospective analyses of cohort studies but used a case–control approach to minimise the number of patients without GDM and, thereby, the cost of adiponectin testing. We would anticipate that wider appreciation of the clinical utility of adiponectin testing and the availability of an automated assay with equivalent performance to current manually intensive ELISAs [50] may enable universal standardised values of adiponectin and decrease the cost of the test, especially as adiponectin can be measured in the same non-fasting samples as routine booking bloods. Given the current existing data sources and the lack of individual participant data, a direct comparison between the predictive accuracy of circulating adiponectin and other biomarkers or a combination of circulating adiponectin with other biomarkers or maternal characteristics was not feasible, but should now be examined in future prospective studies.

### Conclusion and implication for clinical practice and future research

First trimester biomarkers that can predict independently or additively late pregnancy complications have the potential to stratify women in different risk categories, initiate different surveillance patterns and trigger preventative measures. This approach would be complementary to universal screening for GDM at the end of the second trimester, with diagnosis and treatment of milder types of GDM improving perinatal outcomes. Furthermore, in many countries, including the UK, where universal screening is not available, accurate biomarkers can optimise targeted screening by reducing the numbers of false positives and false negatives, with a potential reduction in cost and the clinical implications of missing GDM cases. Current clinical guidelines suggest risk stratification solely based on maternal baseline characteristics and previous history, using a categorical approach rather than a risk score [6]. However, the prognostic characteristics (i.e. sensitivity and specificity) of such an approach are limited [51]: a combination of risk stratification strategies is not associated with a substantial improvement [7] and is lower than the summary prognostic characteristics identified for serum adiponectin. Alternative biomarkers including fasting glucose exhibit poor prognostic characteristics [8, 9, 44]. We acknowledge that the moderate number of pooled studies with different designs and characteristics limit the generalisability of our findings, but they set the foundation for further research. We would therefore propose that risk stratification incorporating simple maternal characteristics, along with selected biomarkers such as adiponectin, could improve the accuracy of risk stratification for GDM. Confirmation of this approach in large contemporary prospective studies, along with health economic evaluation, is warranted in order to evaluate and determine in conjunction with traditional risk factors the incremental value of circulating adiponectin in predicting GDM.

## Electronic supplementary material

ESM Methods (PDF 68 kb)

ESM Fig. 1 (PDF 37 kb)

ESM Fig. 2 (PDF 120 kb)

ESM Table 1 (PDF 323 kb)

ESM Table 2 (PDF 82 kb)

## Abbreviations

DOR: Diagnostic odds ratio
GDM: Gestational diabetes mellitus
HSROC: Hierarchical summary receiver operating characteristic
ROC: Receiver operating characteristic
