# Supplementary material for: Accuracy of circulating adiponectin for predicting gestational diabetes: a systematic review and meta-analysis
Source: Diabetologia. 2016 Jan 14;59:692–9. doi: 10.1007/s00125-015-3855-6 (PMC4779132; doi:10.1007/s00125-015-3855-6)
Supplement: Supplementary file 1 — (PDF 68 kb) [file 125_2015_3855_MOESM1_ESM.pdf]

Literature Search Strategy (references are referring to the main text reference list).

A systematic search of the biomedical databases, using the search terms ‘adiponect* AND gestation* diabetes’ AND ‘pregnan* AND diabetes AND adiponect*’, produced 1095 hits (PubMed, 349; Medline, 76; Embase, 294; Web of Science, 376). After excluding duplicates, 489 citations were identified (Fig 1). Unpublished literature (open grey website or hand searching of references) meeting the search indices was not identified. After excluding articles based in the title or the abstract, 39 articles were assessed fully for eligibility. Twenty six studies were excluded with a reason recorded (Fig 1); thus 13 studies were selected for the systematic review [12-15, 29, 31-38].
