# Supplementary figures and images for: Accuracy of circulating adiponectin for predicting gestational diabetes: a systematic review and meta-analysis
Source: Diabetologia. 2016 Jan 14;59:692–9. doi: 10.1007/s00125-015-3855-6 (PMC4779132; doi:10.1007/s00125-015-3855-6)

**ESM Fig 1:** Quality assessment of the selected studies based on the QUADAS-2 tool.

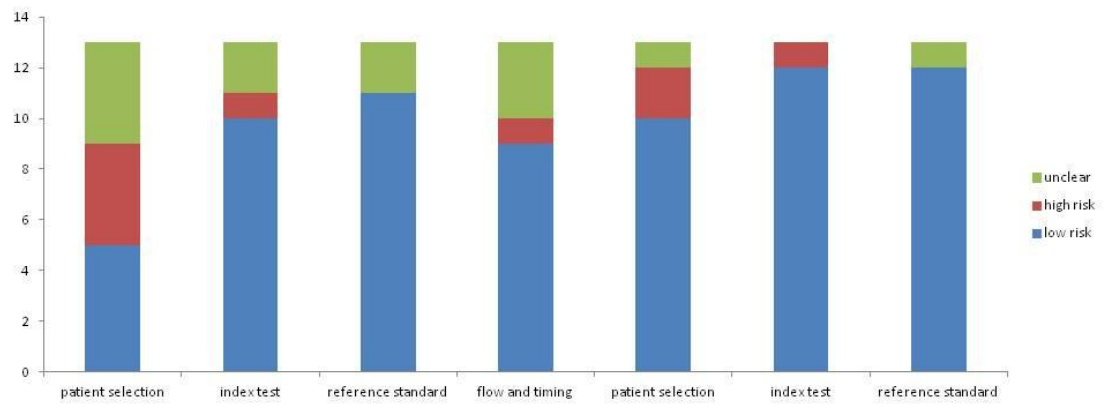

Supplement: Supplementary file 2 — (PDF 37 kb) [file 125_2015_3855_MOESM2_ESM.pdf]

**ESM Fig 2:** Funnel plot of the studies included in the meta-analysis. Diagnostic Odd Ratio (DOR)

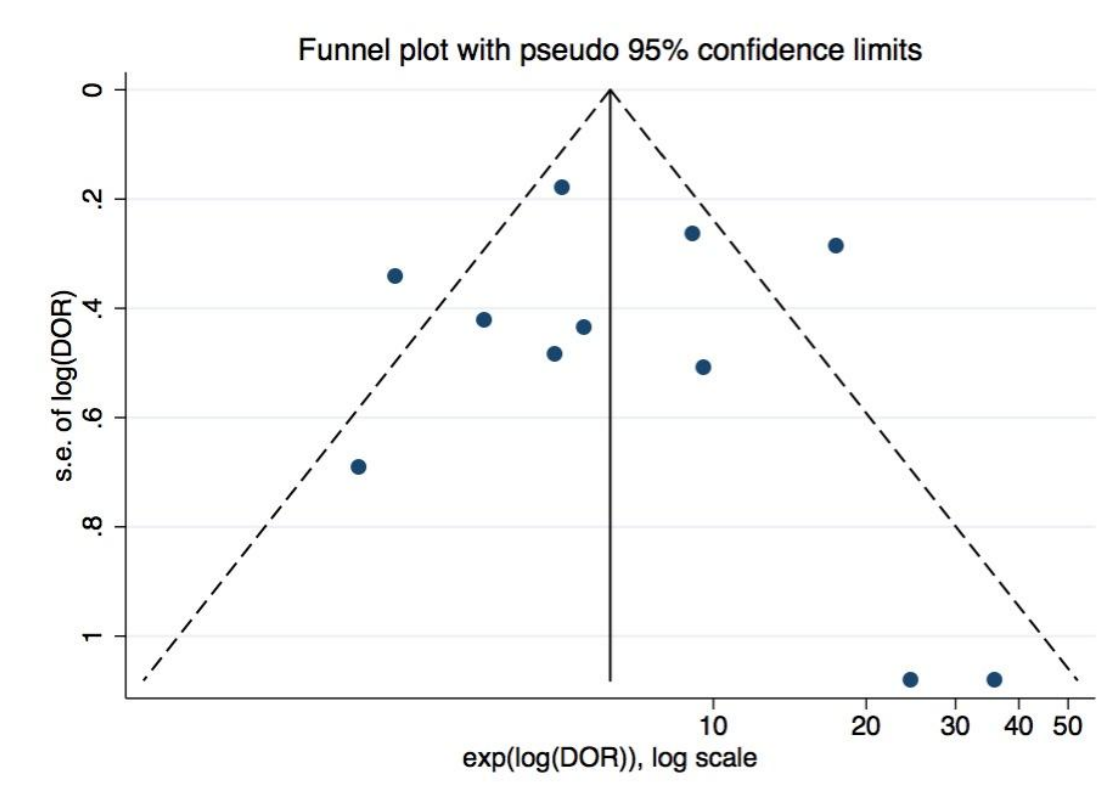

Supplement: Supplementary file 3 — (PDF 120 kb) [file 125_2015_3855_MOESM3_ESM.pdf]
