# Supplementary material for: Accuracy of circulating adiponectin for predicting gestational diabetes: a systematic review and meta-analysis
Source: Diabetologia. 2016 Jan 14;59:692–9. doi: 10.1007/s00125-015-3855-6 (PMC4779132; doi:10.1007/s00125-015-3855-6)
Supplement: Supplementary file 4 — (PDF 323 kb) [file 125_2015_3855_MOESM4_ESM.pdf]

ESM Table 1: Characteristics of the studies included in the systematic review.

Abbreviations (Gestational diabetes mellitus: GDM, intra-uterine growth restriction: IUGR, oral glucose tolerance test: OGTT). Reference numbers are referring to the main text reference list.

Study	Population	Study design	Special study characteristics	Total (N)	GDM (N)	Controls (N)	Adiponectin Measurement	Timing of Adiponectin	Criteria of GDM Diagnosis	Timing of GDM
Williams *et al* 2004 [31]	Participants were selected from the OMEGA cohort study. 1000 participants enrolled in the OMEGA study between 1996-2000. 41 developed GDM, and 70 were randomly selected as controls. Inclusion criteria: All women who initiated antenatal care prior to 16 weeks gestation. Exclusion criteria: Women aged under 18 years, not able to communicate in English, history with pre-existing hypertension or diabetes, non-continuing pregnancies and unknown outcomes	Prospective nested case control study	84% of the study population of White non Hispanic origin	111	41	70	Plasma adiponectin was measured with ELISA (B-Bridge International, Inc., San Jose, CA)	13 weeks gestation	American Diabetes Association criteria (two or more): fasting > 5.3, 1-h > 10.0, 2-h > 8.6; 3-h > 7.8 mmol/L	
Weerakeit *et al* 2006 [32]	Women at high risk of GDM (age>30 years, obesity, family history of diabetes, previous GDM, glycosuria, symptoms of hyperglycaemia, history of poor obstetric outcome) were enrolled between July 2004 and March 2005 in Thailand. Exclusion criteria: women with hypertension or pre-exisiting diabetes, chronic disease or positive tests for syphilis, Hep B, or HIV.	Cohort study	Conducted in Thailand	359	60	299	Standardized immunoassay kit (Linco Research, Inc., St. Charles, MO, USA).	21-27 weeks gestation	American Diabetes Association criteria	24-28 weeks gestation

Gao *et al* 2008 [33]	Blood samples from consecutive women attending antenatal clinic, in Bejing, between January 2004 and March 2005. Exclusion criteria: Women with hypertension, history of GDM, abnormal fasting glucose in 1st trimester or chronic liver or renal disease.	Prospective nested case control study	Chinese origin	42	22	20	ELISA (Yuetai Company, China)	14-20 weeks gestation	75g OGTT (two or more): fasting glucose > 5.8, 1-h > 10.5, 2-h > 9.2, and 3-h > 8.0 mmol/L	24-32 weeks gestation
Lain *et al* 2008 [14]	Nested case control from longitudinal study at Magee-Women's Hospital, Pittsburgh. Nulliparous women were enrolled at <16 weeks gestation at first routine antenatal appointment, between 1997 to 2001. Controls were matched for gestational age at blood sampling and delivery.	Nested case control from longitudinal	Nulliparous women<br>75% of study population of white descent	59	30	29	Total adiponectin was measured with Radioimmunoassay kit from Linco Research Inc. (St Charles, MO)	9.3 +/-2.6 weeks gestation	American Diabetes Association criteria	24-28 weeks gestation
Georgiou *et al* 2008 [12]	Consecutive pregnant women were prospectively recruited and blood sampling was performed at the first antenatal visit and at the time of OGTT. 14 out of 250 women developed GDM. Women with GDM were matched (age, gravidity, parity, ethnicity and BMI) with equal number of controls. Exclusion criteria: Women with pre-exisiting diabetes, or pregnancies complicated by pre-eclampsia, IUGR or fetal macrosomia were excluded.	Matched case control study	12 out of 28 women of Asian descent (6 with GDM and 6 controls). Remaining participants of White descent.	28	14	14	Adiponectin ELISA (R&D Systems, Minneapolis, MN)	11 weeks gestation	75 g glucose load. GDM if fasting glucose $\geq$ 5.5 and/or a 2 –h $\geq$ 8.0 mmol/L.	28 weeks gestation
Paradisi *et al* 2010 [13]	Consecutive Caucasian women with singleton pregnancies at high risk of GDM (obesity, personal history of GDM, glycosuria, family history of diabetes) were recruited. Exclusion criteria: women with hypertension, type 2 diabetes or taking any drugs known to affect lipid or glycaemic metabolism were excluded.	Prospective cohort study	High risk for GDM<br>Caucasian	50	12	38	Human Adiponectin ELISA kit<br>(B-bridge international, Sunnyvale, CA)	7-10 weeks gestation	American Diabetes Association criteria	3rd trimester
Savvidou *et al* 2010 [34]	A nested case control study using first trimester stored samples (<3 years old) from a large prospective observational study conducted at King's College Hospital, London. For each woman who developed GDM, 2 controls were matched. Exclusion criteria: women with pre-existing diabetes or twin pregnancies.	Prospective nested case control	Mixed ethnicity (around 60% of white European descent)	372	124	248	Adiponectin ELISA (R&D Systems, Abingdon, U.K.)	11-13 weeks gestation	WHO criteria	24-28 weeks gestation
Ferreira *et al* 2011 [29]	A nested case control study using first trimester stored samples (<3 years old) from a large prospective observational study conducted at King's College Hospital, London. For each woman who developed GDM, 2 controls were matched. Exclusion criteria: women with pre-existing diabetes or twin pregnancies.	Prospective nested case control	Mixed ethnicity	400	100	300	Adiponectin ELISA (R&D Systems, Abingdon, U.K.)	11-13 weeks gestation	WHO criteria	24-28 weeks gestation

Ianniello *et al* 2013 [35]	Consecutive Caucasian women with singleton pregnancies at high risk of GDM (obesity, personal history of GDM, glycosuria, family history of diabetes) were recruited. Exclusion criteria: women with hypertension, type 2 diabetes or taking any drugs known to affect lipid or glycaemic metabolism were excluded.	Prospective cohort study	High risk for GDM Caucasian	32	16	16	Human Adiponectin (B-bridge international, Sunnyvale, CA)	8-11 weeks gestation	American Diabetes Association criteria	3rd trimester
Lacroix *et al* 2013 [36]	A prospective cohort study of women recruited between 6-13 weeks of pregnancy in Quebec, Canada.<br>Exclusion criteria: Women age under 18 or over 40 years, with multiple pregnancy, pregestational diabetes, drug or alcohol misuse, uncontrolled endocrine disease, renal failure, or other major medical conditions which might affect glucose metabolism	Prospective cohort study	Around 96% of the cohort of white European descent	445	38	407	Total adiponectin radio-immunoassay (Millipore)	6-13 weeks gestation	International Association of Diabetes and Pregnancy Study Groups (IADPSG) criteria	24-28 weeks gestation
Rasanen *et al* 2013 [37]	Participants were retrospectively chosen from the Finnish maternity serum bank. Participants had to have sufficient quantity of first trimester serum collected between 5-13 weeks gestation.<br>Exclusion criteria: multiple gestations and pregnancy complications, including preeclampsia or preterm labour.	Case control study		182	90	92	ELISA with adiponectin monoclonal antibody (840965)	5-13 weeks gestation	75g OGTT: fasting glucose > 5.3 1-h > 10.0, 2-h > 8.6	2nd trimester
Hedderson *et al* 2013 [38]	Women who participated in the Kaiser Permanente Northern California Multiphasic Health Check-up Exam (1984-1996) with a serum sample obtained and a subsequent pregnancy (1984-1996) were selected for the analysis. 2 control subjects were matched to each case, for year of blood draw, age at exam, age at pregnancy, and number of intervening pregnancies.	Nested case control	Mixed ethnicity	753	256	497	Radio-immunassay (Millipore)	Pre-pregnancy	100g 3h OGTT according to the American College of Obstetricians and Gynecologists	
Maitland *et al* 2014 [15]	A pilot trial of a complex intervention of dietary advice and physical activity aimed at improving glucose homeostasis in obese pregnant women, from the multicentre randomised controlled (UPBEAT) trial.	Randomised control trial	Obese women Mixed ethnicity	106	29	77	Adiponectin ELISA (R&D Systems, Abingdon, U.K.)	$15\text{-}17^{+6}$ weeks gestation	International Association of Diabetes and Pregnancy Study Groups (IADPSG) criteria	27-28+6 weeks gestation
