# Supplementary material for: Accuracy of circulating adiponectin for predicting gestational diabetes: a systematic review and meta-analysis
Source: Diabetologia. 2016 Jan 14;59:692–9. doi: 10.1007/s00125-015-3855-6 (PMC4779132; doi:10.1007/s00125-015-3855-6)
Supplement: Supplementary file 5 — (PDF 82 kb) [file 125_2015_3855_MOESM5_ESM.pdf]

ESM Table 2: Negative Predictive Values (NPV) and Positive Predictive Values (PPV) for different prevalence points of Gestational Diabetes (GDM).

Prevalence (%)	NPV (%)*	PPV (%)*
5	97.7	13.3
10	95.2	24.5
15	92.6	34.0
20	89.8	42.1

* Estimated sensitivity of 64·7 % and specificity of 77.8 % from meta-analysis.

NPV= specificity x (1-prevalence)/ [(1-sensitivity) x prevalence + specificity x (1-prevalence)]

PPV= sensitivity x prevalence/ [sensitivity x prevalence + (1-sensitivity) x (1-prevalence)]
